# Stimulated Emission Depletion Inspired Sub-100 nm Structuring of Epoxides Using 2-Chlorothioxanthone as Photosensitizer

**DOI:** 10.1021/acsomega.4c00031

**Published:** 2024-04-18

**Authors:** Sourav Islam, Thomas A. Klar

**Affiliations:** Institute of Applied Physics, Johannes Kepler University Linz, 4040 Linz, Austria

## Abstract

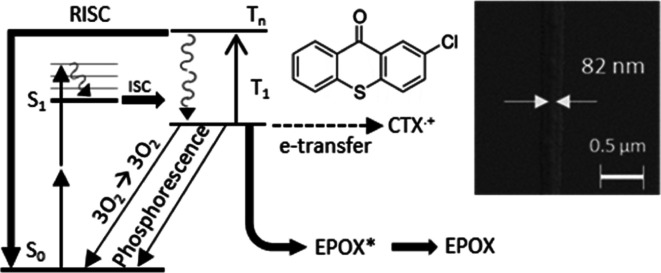

Until very recently, the enhancement of multiphoton-based optical lithography by stimulated emission depletion (STED) inspired techniques was limited mostly to (meth)acrylates. Epoxides, which play an important role in semiconductor clean-room technology, were basically excluded from capitalizing on STED-inspired lithography, and if they were successfully used in STED-inspired lithography, the achievable structure sizes remained at 125 nm and above. We now found that using 2-chlorothioxanthone (CTX) as a sensitizer for a sulfonium salt acting as the photoinitiator allows for shrinking the structure size down to 83 nm. Compared to the previously used sensitizer 2-isopropylthioxanthone, the triplet lifetime of CTX within the epoxide monomers is supposed to be prolonged by 40%, which renders an optical depletion via excited triplet state absorption more efficient, leading to a sub-100 nm structuring capability.

## 1 Introduction

Multiphoton lithography (MPL) enjoys a variety of advantages over standard UV lithography such as inherent three dimensionality and lower photon energy.^1^ But it suffers from comparatively long wavelengths in the visible or near IR range, limiting the minimally achievable dimensions. Stimulated emission depletion (STED) lithography and related concepts have brought a significant improvement in this field.^2−7^ The genesis of STED can be traced back to the 1990s, when theoretical prediction^8^ and experimental demonstration^9^ brought this tool to life and took fluorescence microscopy beyond the diffraction limit. At the core of STED microscopy lies turning fluorescence off in the outer rim of the focal point spread function (PSF) by introducing a donut-shaped second beam known as the “depletion beam”. Very soon, it was realized that STED can be taken beyond fluorescence microscopy: it can also be used to spatially control radical photoinitiators. Since then exceptional achievements have been reported for writing (meth)acrylate structures with deep subwavelength feature sizes and with diffraction-unlimited resolution.^7,10−12^ Most of these works were carried out for the free radical polymerization of (meth)acrylate monomers. Almost no focus has been devoted to transferring the knowledge to the cationic polymerization of epoxide-based resins. However, epoxides enjoy some distinct advantages compared with acrylate-based resins. These include lower cytotoxicity, lower shrinkage yielding lower residual stress in the cured materials, better adhesion properties on various substrates, little oxygen-inhibition relaxing the necessity of an inert atmosphere during curing, rapid curing, nonpollutant and solvent-free formulations, or biocompatibility. All of these make epoxy-based resins the standard workhorse in the semiconductor industry and in many other fields.^13^

Despite all these advantages, STED-inspired, diffraction-unlimited nanolithography of epoxides was shown only recently.^14^ The resin used in ref (14) comprised 3,4-epoxycyclohexylmethyl 3,4-epoxycyclohexanecarboxylate (EPOX) as the monomer, 1 wt % triarylsulfonium hexafluoroantimonate (AR_3_S/SbF_6_) as initiator, and 4 wt % of 2-isopropylthioxanthone (ITX) as a sensitizer. A robust theoretical framework was proposed and subdiffractional lithography was shown by writing 125 nm wide features.^14^ Without the depletion, i.e., with MPL alone, the line width was 370 nm, typical for MPL lithography of epoxides.^15,16^ Although this was an improvement of 66%, the 100 nm barrier was not undercut. In the following, we will show how to reach feature sizes down to 83 nm. This is a substantial improvement and the first time that epoxides have been structured below 100 nm by STED-inspired lithography. The key point was to exchange the 4 wt % of the ITX sensitizer by 4 wt % of 2-chlorothioxanthone (CTX).

## 2 Basic Concept

**Figure 1:**
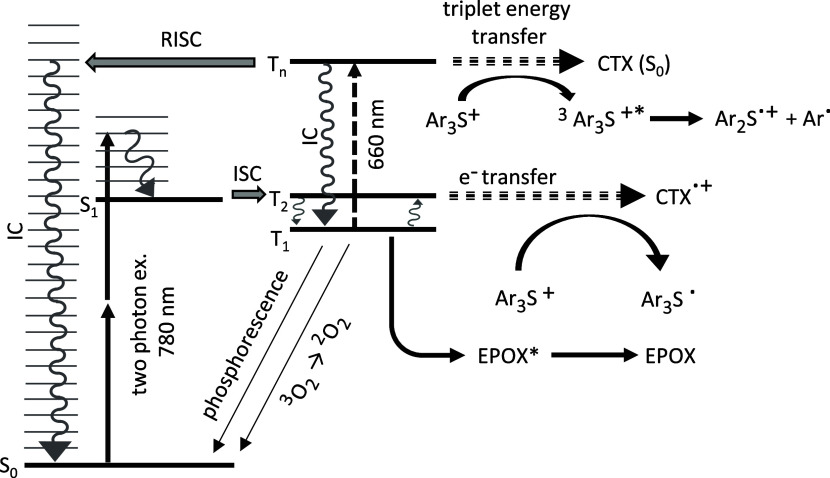
Jabłonski diagram of a TX sensitizer, e.g., CTX. After 780 nm two-photon excitation, the TX undergoes rapid inter-system crossing (ISC) to the triplet system, from where it can excite the onium Ar_3_S^+^ via electron transfer, while it turns itself into a CTX^•+^ radical cation. Alternative decay channels of the *T*_1_ are phosphorescence, oxygen excitation, or energy transfer to EPOX (not leading to polymerization). If additional 660 nm light is provided, CTX is further excited to *T*_*n*_, from where it has several options, such as falling back to *T*_1_ via internal conversion (IC), undergoing reverse intersystem crossing (RISC) followed by fast IC down to *S*_0_, and finally transferring its energy to the Ar_3_S^+^. Further details are described in the text.

STED-inspired nanolithography of epoxides does not rely on “true” STED but rather on a depletion mechanism based on transient triplet state absorption by a thioxanthone (TX) sensitizer (CTX or ITX).^4,17−19^ This mechanism is abbreviated transient state absorption depletion (TAD), meaning triplet state (or transient state) absorption depletion.^20^Figure 1 shows the key molecular dynamics in the initiator/sensitizer system.^14^ In a typical TX, the lowest triplet state *T*_1_ is quickly reached after two-photon excitation via rapid ISC.^21−26^ From there, there are several pathways available. For instance, cationic polymerization can be initiated via a thermal population of the *T*_2_ state, followed by a rapid transfer of an electron from the sensitizing CTX toward the aryl-sulfonium cation.^27−30^ This yields a CTX^•+^ radical cation, which induces epoxide polymerization. The rate of this (wanted) decay channel via electron transfer is determined by the concentration of Ar_3_S^+^. The molar quenching rate via electron transfer was found to be *k*_el.T_ = 210 M^–1^ μs^–1^ for TXs.^30^ With a concentration of Ar_3_S^+^ units of 10.73 mM, we end up with γ_el.T_ = 2.25 μs^–1^. This is the wanted decay channel in the case of MPL, as it initiates the polymerization. However, there are several other competing decay channels of the *T*_1_. There are direct transitions (for instance by phosphorescence) back to the *S*_0_, with a typical rate of γ_0_ = 0.1 μs^–1^.^27^ Background triplet oxygen may react with the triplet TX with a typical rate of γ_Ox_ = 4 μs^–1^.^27^ And finally, there is a substantial quenching of the *T*_1_ by electron transfer to an EPOX monomer. This leads to a transiently excited EPOX* monomer which, however, cannot start a polymerization reaction and, hence, the excitation is lost for polymerization.^27^ Manivannan and Fouassier found that the molar quenching coefficient by EPOX is different for ITX and CTX, namely, 6.0 and 4.0 M^–1^ μs^–1^, respectively. With an EPOX (being the monomer and the solvent) concentration of 4.41 M, one ends up with quenching rates by the monomers γ_EPOX_ of 26.5 or 17.6 μs^–1^ for ITX or CTX, respectively. For the time being, we do not consider any triplet–triplet excitation (we will later add such an excitation using a 660 nm TAD laser). The total decay rate of the *T*_1_ is then given by
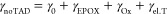
1

Using the above numbers for the individual rates, γ_noTAD_ yields 33 or 24 μs^–1^ for ITX or CTX, respectively, corresponding to *T*_1_ lifetimes of τ_T1_ of 30 and 42 ns, respectively. This 40% longer lifetime of the transient triplet state *T*_1_ of CTX in EPOX compared to that of ITX is the key reason for improved TAD lithography, as outlined further down.

We now include the transient absorption of 660 nm light in our discussion. This excites the TX further from *T*_1_ to *T*_*n*_. From there, it can either undergo IC back to *T*_1_ with a typical rate of γ_IC_ = (0.4 ps)^−1^,^14,26^ or undergo RISC (Figure 1), followed by quick IC within the singlet system. This brings the TX back to the *S*_0_ and will lead to an effective TAD process.^14^ This is the same process as for subdiffractional STED-inspired lithography of acrylates using ITX as a two-photon radical starter.^17−19,31,32^ Assuming that the rates of RISC and ISC are similar, we end up with γ_RISC_ = (7 ps)^−1^.^14,24,26,31^ A third process can also lead to a depletion of *T*_*n*_: in the *T*_*n*_ state, a TX has sufficient energy for triplet energy transfer to the Ar_3_S^+^.^30,33^ The TX turns from the *T*_*n*_ down to the *S*_0_ and simultaneously, the Ar_3_S^+^ is excited to its triplet state, from where it decays into an Ar^•^ cation and an Ar_2_S^•+^ radical cation which initiates polymerization. A typical rate for energy transfer is in the range of γ_ET_ = (500 ps)^−1^, 2 orders of magnitude lower than RISC and 3 orders of magnitude lower than intratriplet IC, so we will neglect this possibility for the time being.

The optical depletion via the 660 nm laser is effective if RISC from *T*_*n*_ outperforms the electron transfer from the lower triplet states to Ar_3_S^+^. At first sight, this seems unlikely, as γ_IC_ > γ_RISC_, but, as we have detailed in ref (14), it is indeed possible if the 660 nm power is strong enough so that the 660 nm laser effectively cycles the TX molecule between *T*_1_ and *T*_*n*_ until it eventually escapes via RISC. To render TAD efficient, this must happen, on average, before the electron transfer takes place. Hence, the 660 nm laser has to be sufficiently powerful. A useful number, which provides a quantitative understanding of the necessary depletion power, is the so-called saturation power of depletion, *P*_sat_, where the 660 nm laser depletes the electron transfer down to 1/*e*, whereby *e* is Euler’s number. We have recently shown that^14^
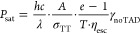
2

Hereby, λ is the wavelength of the TAD laser, *c* is the speed of light, *h* is Planck’s constant, *A* is the area of the PSF of the depleting beam, σ_TT_ is the transient absorption cross section, with a typical value of σ_TT_ = 3.9×10^–17^ cm^2^ in case of TXs,^14^*T* = 0.9 is the transmission of the objective lens at 660 nm, and η_esc_ = γ_RISC_/(γ_RISC_ + γ_IC_) = 5.4% is the escape efficiency that a TX molecule in the *T*_*n*_ escapes the triplet system by RISC rather than falling down to *T*_1_ by IC.

The important conclusion from these considerations is that TAD is most efficient if already a low power of 660 nm light depletes the electron transfer effectively, which means that *P*_sat_ should be as small as possible; in other words, γ_noTAD_ shall be small, which is equivalent to a long triplet lifetime τ_T1_ = 1/γ_noTAD_. Even without eq 2, this is intuitively clear because a long triplet lifetime makes it easier for the depletion beam to effectively deplete the triplet system and hence prevent polymerization. Putting everything together, we expect that CTX, used as sensitizer in this study, should be more easily depletable than ITX and render the smaller feature sizes, because the theoretical triplet lifetimes were found to be 30 and 42 ns in the case of ITX and CTX, respectively. Compared to our previous study with ITX, where a theoretical value of *P*_sat_ = 3.4 mW was found,^14^ we now retrieve *P*_sat_ = 2.4 mW when putting all numbers for CTX into eq 2.

## 3 Experimental Methods

4 wt % amount of CTX and 1 wt % of (triarylsulfonium hexafluoroantimonate (AR_3_S/SbF_6_) of 50 wt % in propylene carbonate, Sigma-Aldrich prod. no. 654027) were added to EPOX and mixed according to the following routine: (i) stir the mixture with 500 rpm for 30 min at room temperature (RT), (ii) sonicate it at 60 °C for 15 min, and (iii) stir for another 30 min at RT. Steps (i–iii) were repeated (typically 3–4 times) until a clean resin without any undissolved CTX was achieved.

All components were purchased from Sigma-Aldrich and used as received. The home-built lithography setup was described in detail elsewhere.^6,14^ In brief: a pulsed laser of 780 nm wavelength (82 MHz repetition rate, 110 fs, FFS-tSHG, Toptica, Germany) was used for two-photon excitation of the CTX. To enhance attachment of the structures to the substrate, glass slides of thickness 170 ± 5 μm (Marienfeld GmbH, Germany) were coated with (3-glycidyloxypropyl) trimethoxysilane (Sigma-Aldrich) prior to the application of the resin. Then, a glass slide was mounted on a three-axis piezo stage (P-562.3CD, Physik Instrumente (PI), Germany) with a bidirectional positioning accuracy of 2/2/4 nm (*x*/*y*/*z*). A continuous wave (CW) laser of wavelength 660 nm (Opus, Laser Quantum, Germany) was used to induce TAD. All powers were measured at the back aperture of the objective lens (60×, NA 1.49, oil immersion, Olympus, Japan). The writing speed during fabrication was 2 μm/s. After fabrication, the sample was developed by very carefully drop-casting 5–10 drops of ethanol until the glass slide was visibly clean. To measure the line heights, we used atomic force microscopy (AFM) (CP-II, Digital Instruments, USA). The line widths were measured by scanning electron microscopy (SEM) (Zeiss 1540XB) after evaporating approximately 10 nm of gold.

## 4 Results and Discussion

**Figure 2:**
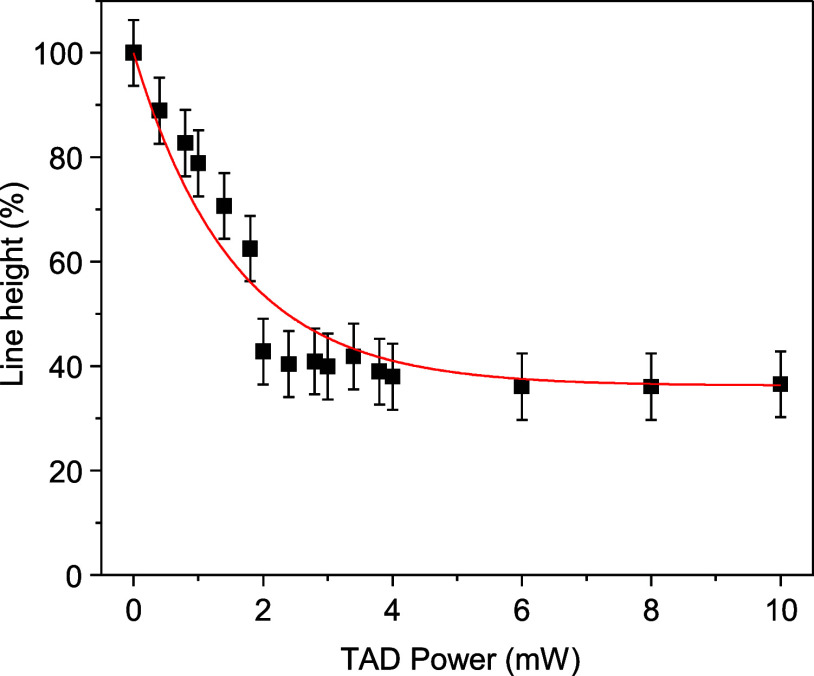
Suppression of polymerization: line height (measured with AFM) as a function of TAD power. The excitation power was 3.3 mW. The red line is a fitted single-exponential decay. The residuum is 36%, and the 1/*e* exponential decay power is *P*_sat_ = 1.5 ± 0.1 mW.

In the first experiment, we wrote lines on a glass substrate. Without the depletion laser, the lines were typically 210 nm high and about 300 nm wide, similar to our previous result with 4 wt % ITX as sensitizer instead of 4 wt % CTX.^14^ This shows that ITX and CTX are interchangeable in terms of the MPL performance. We further note that it is well known in the literature that the heights of lines written directly on a glass substrate with MPL can be well below the axial size of the PSF,^34^ and that a line width of 300 nm is typical for MPL of epoxides.^15,16^ Subsequently, we gradually increased the depleting TAD power, confocalized with an ordinary PSF on top of the MPL excitation focus. Figure 2 shows the relative reduction in line height as a function of the TAD power. A roughly exponential decrease in line height with increasing TAD power is observed as illustrated by the red line, which is an exponential fit with a decay constant of *P*_sat_ = 1.5 ± 0.1 mW. This is a 12% reduction compared to the previously obtained experimental result of *P*_sat_ = 1.7 mW in the case of 4 wt % ITX as the sensitizer.^14^ Although this is less reduction than theoretically expected, CTX turns out to be the more efficient sensitizer for TAD lithography of EPOX than ITX. The line width does not change as a function of TAD power when an ordinarily shaped PSF is used, as expected (Figure S1).

The saturation power determined by fitting an exponential decay (or theoretically by eq 2) plays a role in TAD lithography similar to that of the so-called saturation power *P*_sat_ in STED microscopy, which is defined as the depletion power where the fluorescence is quenched down to 1/*e* of its original value without depletion. It has been shown that the minimally achievable resolution in STED microscopy as a function of the applied depletion power *P*_depl_ is given by^35^
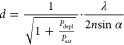
3whereby λ is the vacuum wavelength of light, *n* is the refractive index of the immersion medium, and α is the half-opening angle of the objective lens. In principle, one could achieve infinitely small resolution in STED microscopy simply by increasing *P*_depl_ ad infinitum, regardless of the specific value of *P*_sat_. However, in practice, the maximally tolerable *P*_depl_ is limited by the bleaching of the dye molecules. Consequently, a low *P*_sat_ is important for high resolution in STED microscopy.^36^ Analogously in STED-inspired lithography, the maximally applicable *P*_depl_ is limited by microexplosions and, in the case of epoxides, also by the background polymerization due to energy transfer (Figure 1). The smaller saturation power in the case of CTX compared to ITX should therefore result in reduced structure sizes.

**Figure 3:**
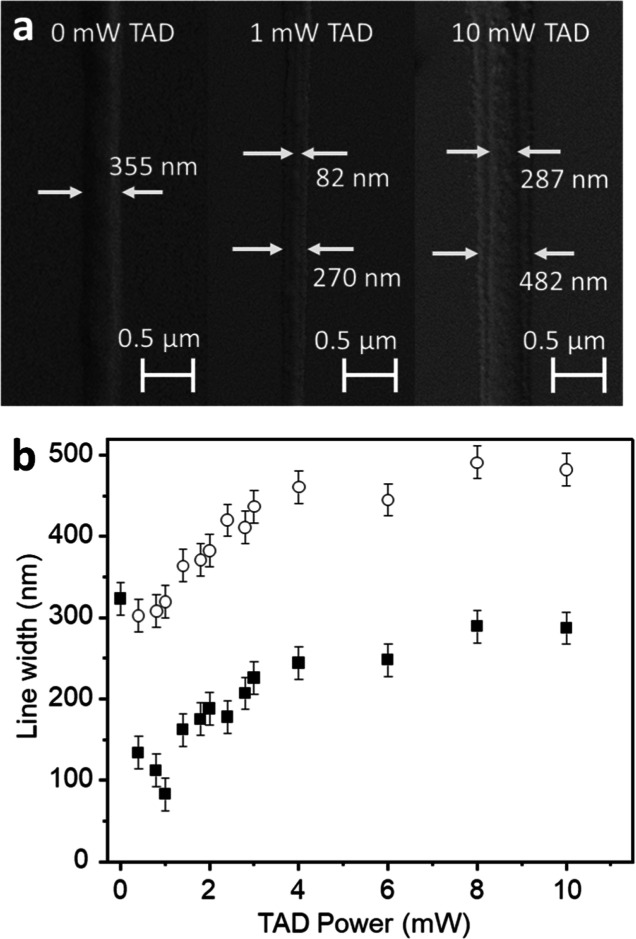
(a) Lines written with 3.3 mW of 780 nm excitation power and different 660 nm TAD powers as indicated. The pure MPL line width is 355 nm (0 mW TAD). Applying 1 mW of TAD, the central line narrows by 77% down to 82 nm, but a faint pedestal of 270 nm width is visible. In the case of 10 mW, the central line is 287 nm, and the pedestal widens to 482 nm. (b) Widths of the central lines (filled squares) and the pedestals (open circles) as a function of TAD power. Data points in (b) are averages of up to 4 independent experiments.

We proceeded to test the achievable minimal feature sizes by switching from an ordinary PSF to a donut-shaped PSF by introducing a 2π phase spiral in the beam path of the 660 nm depletion beam. After the lines were developed, they were overcoated with 10 nm of gold and imaged in an SEM. Figure 3a shows SEM images of lines written with a 3.3 mW excitation power for three different TAD powers. At 0 mW TAD power, which means pure MPL, the achieved line width is 355 nm. At 1 mW TAD power, the width of this particular line measures 82 nm, which means that the feature size shrank by 77%. In addition, a faint pedestal of 270 nm appears below the line, similar to our previous results with ITX. We refer this pedestal being linked to the undepletable residuum (Figure 2) due to energy transfer from the *T*_*n*_ to the onium (upper right part of Figure 1).^14^ At 10 mW TAD power, the line in the middle and the pedestal both broaden to 287 and 482 nm, respectively. The experiment was repeated four times to ensure the repeatability of the results. The feature sizes were measured for each single line at four different positions, and an average was determined for that line. Then, these averaged widths from up to four lines (at the same TAD power) were averaged and are plotted in Figure 3b. In some cases, lines did not survive the washing step and, hence, there are some TAD powers, where the average is taken over less than four individual lines. Tables S1 and S2 in the Supporting Information provide the widths of all individual lines and the averaged line widths for each TAD power. Figure 3b shows a plot of those averaged feature sizes as a function of the TAD power. The error bars of ±20 nm are estimated from the run-to-run variations of the measured feature sizes. The full squares represent the widths of the central line and the open circles represent the widths of the pedestals. The central line widths rapidly decrease with increasing TAD powers up to 1 mW, from whereon the line widths widen again. The minimally achieved average feature size is 83 nm. This is, to the best of our knowledge, the first experimental demonstration of a sub-100 nm feature width in STED-inspired cationic photoinhibition lithography. The width of the pedestals increases with increasing TAD powers, consistent with our previous report.^14^

In Table 1, we compare the achieved minimal feature sizes and saturation powers of this study with our previous results. In all cases, EPOX was the monomer, excitation was performed with approximately 3.2–3.3 mW of 780 nm, 120 fs laser, and a CW laser of 660 nm wavelength was used for depletion. A clear correlation between *P*_sat_ and the minimally achieved feature size is obvious. Further, *P*_min_ reflects the TAD powers at which minimal feature sizes were achieved. Beyond these TAD powers, the features widen again (cf., Figure 2). It is remarkable that *P*_sat_ and *P*_min_ are similar; however, *P*_min_ is systematically smaller than *P*_sat_.

**Table 1:** Comparison of the Saturation Intensities*P*_sat_, the Achieved Minimal Feature Widths, and the TAD Power*P*_min_ at Which These Minimal Feature Sizes Were Achieved for Different Sensitizer/Onium Systems

sensitizer/onium	*P*_sat_ [mW]	minimal feature [nm]	*P*_min_ [mW]	ref
4 wt % CTX, 1 wt % Ar_3_S/SbF_6_	1.5	83	1.0	this work
4 wt % ITX, 1 wt % Ar_3_S/SbF_6_	1.7	125	1.5	(14)
4 wt % ITX, 1 wt % Ph_2_I/PF_6_	8.1	220	6	(14)

## 5 Conclusions

We have demonstrated, for the first time, structure sizes below 100 nm in STED-inspired cationic lithography with epoxide polymers. We have used CTX as the photosensitizer, a sulfonium salt as the photoinitiator, and an epoxide monomer. We have achieved feature sizes of 83 nm, which is a 77% improvement over regular MPL lines and a 33% improvement over the recently achieved epoxide feature sizes using ITX as a sensitizer in STED-inspired nanolithography. We ascribe this improvement to the longer triplet lifetime τ_T1_ of the transient CTX. The longer τ_T1_, the easier it is for the depleting beam to excite the sensitizing TX to a higher triplet state *T*_*n*_, from where it returns to the ground state *S*_0_ via reverse ISC. Consequently, TAD lithography is more efficient with CTX as a sensitizer than with ITX. The fact that our experimental results follow the theoretical predictions for improved depletability corroborates our model of the photophysics of STED-inspired TAD nanolithography. This poses a clear strategy for future improvements of subdiffractional STED-inspired nanolithography of epoxides, namely to search for alternative sensitizers of onium salts which bear intermediate states of long lifetime, similar to STED-inspired RESOLFT microscopy, where intermediate states of a long lifetime lead to low depletion powers and ultrahigh resolution.^36^ An issue that remains to be solved is the avoidance of the undepletable residuum. A possible strategy could be to find a sensitizer which does not excite the onium salt from the high-lying *T*_*n*_ state. Another issue is that no three-dimensional structures such as suspended lines have been written so far. Monomers other than EPOX may solve the problem, specifically when bearing more than two epoxide groups for better cross-linking.

## Supporting Information Available

Feature sizes as a function of TAD power, of up to four different experiments, that lead to the data presented in Figure 3 and width of the lines as a function of TAD power when using an ordinary PSF (PDF)
