# Supplementary material for: Stimulated Emission Depletion Inspired Sub-100 nm Structuring of Epoxides Using 2-Chlorothioxanthone as Photosensitizer
Source: ACS Omega. 2024 Apr 18;9(17):19203–8. doi: 10.1021/acsomega.4c00031 (PMC11064169; doi:10.1021/acsomega.4c00031)
Supplement: Supplementary file 1 — ao4c00031_si_001.pdf [file ao4c00031_si_001.pdf]

Stimulated Emission Depletion Inspired Sub-100 nm Structuring of Epoxides Using 2-Chlorothioxanthone as Photosensitizer

Sourav Islam[1] and Thomas A. Klar[1,*]

[1]Institute of Applied Physics, Johannes Kepler University Linz, 4040 Linz, Austria

* thomas.klar@jku.at

Supplementary Information

A) Data for Figure 3b

Figure 3b in the main text shows averaged line widths of the inner lines and the pedestals as a function of TAD power. The average was taken over four individual experiments. In each experiment, lines are written with different TAD powers. In some cases, the lines detached during development and hence, no data could be obtained in these cases. For those lines which survived development, the width of the inner line and the width of the pedestal was determined from the SEM images at four different positions, each. These four results for each line width were averaged to yield the widths of the inner line and the pedestal. The results are shown in Tables S1 and S2 for the inner lines and the pedestals, respectively, for each of the four experimental runs. Missing entries refer to missing results due to washed away lines. Finally, the widths form these up to four lines per TAD power were averaged and are indicated in the tables S1 and S2 as “average inside width” and “average outside width”, respectively. These are the widths which are displayed as a function of TAD power in Figure 3b in the main text.

TAD Power (mW)	batch 1 (nm)	batch 2 (nm)	batch 3 (nm)	batch 4 (nm)	average inside width (nm)
0	355	312	263	361	323
0.4	143	150	90	152	134
0.8	98	117	101	132	112
1	82			84	83
1.4	149	132	246	122	162
1.8	268	150	146	138	175
2		175	235	153	188
2.4			178		178
2.8	171	217	226	213	207
3		222	239	217	226
4		235	253		244
6	175	245	294	278	248
8	370	215	342	228	289
10				287	287

Table S1: Central line widths for different TAD powers from up to 4 lines from four different experimental runs. Missing data reflects lines that did not survive the washing steps.

TAD Power (mW)	batch 1 (nm)	bacth 2 (nm)	batch 3 (nm)	batch 4 (nm)	average outside width (nm)
0	355	312	263	361	323
0.4	317	310	299	282	302
0.8	250	285	330	368	308
1	270			369	320
1.4	365	287	444	359	364
1.8	365	317	436	365	371
2		360	413	376	383
2.4			420		420
2.8	312	435	483	415	411
3		442	438	430	437
4		447	475		461
6	382	440	515	442	445
8	552	435	526	451	491
10				482	482

Table S2: Outer widths of the pedestals for different TAD powers from up to 4 lines from four different experimental runs. Missing data reflects lines that did not survive the washing steps.

B) Lateral feature widths as a function of TAD power when using an ordinary TAD-PSF

While Figure 2 shows the line height as a function of TAD power when an ordinary PSF is used for the TAD beam (measured with AFM), we here show the lateral feature sizes (measured with SEM).

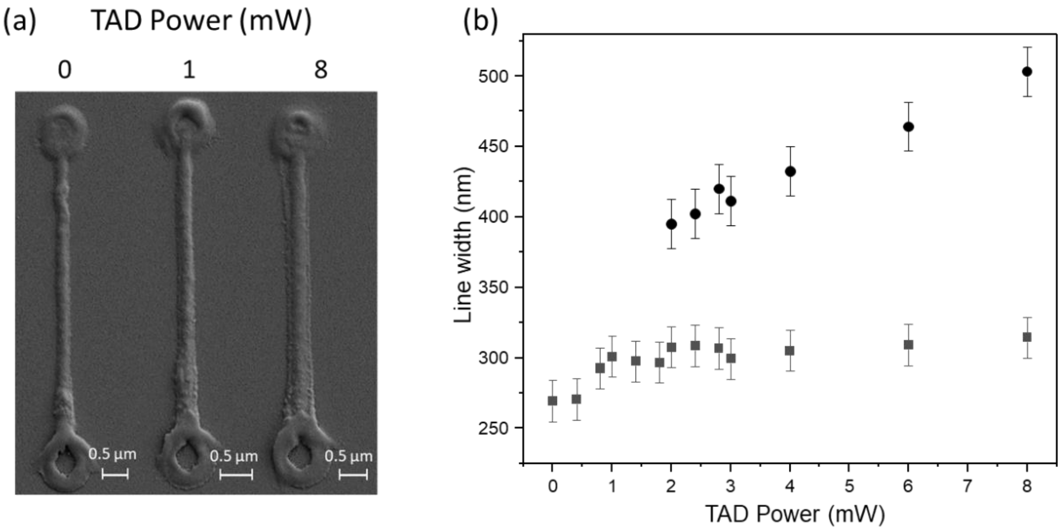

Figure S1: *Lines written with confocalized ordinary point spread functions of 780 nm (3.3 mW) and 660 nm TAD with powers as indicated. The starter comprised 4 wt % CTX and 1 wt %* $Ar_3S{:}SbF_6$*. (a) Lines with 0, 1, and 8 mW of TAD power. (b) Width of the central line (full squares) and the pedestal (full circles), averaged over 3 batch of data, as a function of TAD power. The width of the central line stays essentially the same. From 2 mW onwards, a pedestal appears which keeps on growing in width with increasing TAD power.*
